# Association between empirically driven dietary patterns and cardiometabolic disease risk factors: a cross-sectional analysis in disease-free adults

**DOI:** 10.1186/s12986-025-00965-6

**Published:** 2025-07-09

**Authors:** Arife Yilmaz, Michelle Weech, Vasiliki Bountziouka, Kim G. Jackson, Julie A. Lovegrove

**Affiliations:** 1 Hugh Sinclair Unit of Human Nutrition, Institute of Food, Nutrition and Health, Department of Food and Nutritional Science, Institute for Cardiovascular and Metabolic Research, University of Reading, Reading, RG6 6DZ UK; 2 Computer Simulations, Genomics and Data Analysis Laboratory, Department of Food Science and Nutrition, University of the Aegean, Mytilene, Greece; 3 Great Ormond Street Institute of Child Health, University College London, London, UK; 4 Cardiovascular Research Centre, Department of Cardiovascular Science, University of Leicester, Leicester, UK; 5 Department of Food Engineering, Rafet Kayıs Engineering Faculty, Alanya Alaaddin Keykubat University, Antalya, 07425 Turkey

**Keywords:** Anthropometrics, A posteriori analysis, Diet quality, Lipid profile, Nutrient intake, Principal component analysis

## Abstract

**Background:**

Although links between dietary patterns (DPs) and cardiometabolic disease (CMD) risk markers have been identified in UK populations, these studies often rely on less quantitative measures of dietary assessment and include only a limited number of risk markers.

**Objective:**

This cross-sectional analysis aimed to identify DPs in self-reported disease-free adults using weighed diet diaries and explore relationships with a broad range of CMD risk factors and diet quality.

**Methods:**

Data were collated from five studies conducted in adults living in the UK (2009-2019) and DPs were a posteriori extracted from habitual dietary intake data using principal component analysis. Associations between quartiles (Q) of adherence to the DPs with CMD risk markers, nutrient intakes and the Alternative Healthy Eating Index (AHEI-2010) were evaluated using ANCOVA.

**Results:**

In our cohort [*n* = 646, 58.4% female, mean (SD) age 44 (14) years, and body mass index (BMI) 25.2 (4.0) kg/m^2^] two DPs explained 12% of the variance in habitual food intake. Highest adherence to DP1 (Q4), characterised by diets rich in fermented dairy, fruits, vegetables, wholegrains, nuts/seeds, unsaturated fats/oils and milk and lower in red meat dishes and processed meat, was associated with a lower BMI, waist circumference, diastolic blood pressure, fasting triacylglycerol, non-high-density lipoprotein-cholesterol, remnant-like particle-cholesterol, and total cholesterol:HDL-C ratio and a higher HDL-C and AHEI-2010 score versus Q1 (all *P* ≤ 0.006). In contrast, Q4 vs Q1 of DP2, high in refined carbohydrates, milk and unsaturated fats/oils and low in cruciferous vegetables/spinach, and nuts/seeds, was only associated with a lower HDL-C (*P* = 0.006) and AHEI-2010 score (*P* < 0.001).

**Conclusions:**

In disease-free adults, greater adherence to DP1, which broadly aligned with UK food-based dietary guidelines, was favourably associated with diet quality and CMD risk markers. Our findings could contribute to the evidence base for future food-based dietary recommendations, particularly highlighting the importance of fermented dairy foods.

**Supplementary Information:**

The online version contains supplementary material available at 10.1186/s12986-025-00965-6.

## Introduction

Diet is a key modifiable risk factor in the development and progression of cardiometabolic diseases (CMD), the leading causes of mortality and morbidity in the UK. While existing epidemiological studies have predominantly focused on the impact of specific dietary constituents such as saturated fat (SFA) and dietary fibre intakes, human dietary patterns (DPs) are shaped by a complex interplay of diverse foods and food groups, which may have synergistic or antagonistic effects on health outcomes when consumed together [1]. Principal component analysis (PCA) is a widely used method in epidemiological studies to reduce dimensionality of dietary intake data, extracting exploratory components of highly correlated food groups that can be more easily interpreted as DPs [2]. Empirical data-driven approaches have been used to explore associations between overall dietary intake with health outcomes in different population groups. A meta-analysis of twenty-two observational studies reported strong associations between DPs and cardiovascular disease (CVD) mortality and incidence. In particular, highest adherence to a prudent/healthy DP, rich in plant-based foods, including fresh fruits and vegetables, wholegrains, legumes, seeds, and nuts, and a lower consumption of animal-based foods, was associated with lower pooled relative risk of CVD compared to lowest adherence [3]. However, most studies included in the meta-analysis were performed in non-UK populations, which makes it difficult to translate findings due to the varying dietary assessment methods and habitual dietary intakes between cohorts.

While the dietary data from several large UK cohorts have investigated DPs in relation to CMD risk factors [4–13], these have often used less quantitative measures such as food frequency questionnaires [14]. This may limit the reliability and consistency of the DPs identified and their subsequent relationships with study outcomes. Although links between DPs and CMD risk markers have been identified in UK populations, very few have included a broad range of markers within the same cohort [6, 7, 11]. In the current cross-sectional analysis, we used weighed diet diaries to identify common DPs in self-reported disease-free UK adults (18-70 years) using PCA and explored their associations with a broad range of CMD risk factors. This data-driven approach was used to provide a greater insight into the overall diet of our cohort. The secondary aim was to determine relationships between the identified DPs with nutrient intake, and diet quality (assessed using the Alternative Healthy Eating Index 2010 (AHEI-2010)). We hypothesised that greater adherence to a more healthful DP will be associated with a higher diet quality score and a lower CMD risk.

## Methods

### Study design and population

This cross-sectional analysis employed baseline data from five human studies conducted at the Hugh Sinclair Unit of Human Nutrition at the University of Reading (UK) from 2009 to 2019. The specific details of these studies have been described previously [15–19], with a summary of study designs and participants presented in Supplementary Table 1. All of the studies received a favourable ethical opinion from the University of Reading Research Ethics Committee and written informed consent was obtained prior to study participation. At the time of the baseline visit all participants were following their habitual diet and had not commenced the relevant study intervention. All participants were self-reported disease-free adults aged between 18 and 70 years, residing in Reading and surrounding areas. Common exclusion criteria across the combined studies included a history of myocardial infarction or stroke within the past year, diabetes or other endocrine disorders, bowel disease, cholestatic liver disease, pancreatitis, cancer, use of medications for hyperlipidaemia, hypertension, inflammation, or hypercoagulation, adherence to a weight-loss diet, and excessive alcohol consumption (> 14 units/week). Female participants were also excluded if they were breastfeeding, pregnant, or planning a pregnancy within the timeframe of the original study intervention.

Some notable differences in participant characteristics are as follows: the Dietary Intervention and VAScular function (DIVAS) [18] and REplacement of SaturatEd fat in dairy on Total cholesterol (RESET) [19] studies enrolled males and females who had a moderate risk of CVD (1.5 times higher than that observed in the general population as determined by the Framingham risk score), the DIVAS-2 [17] study included postmenopausal women only, while the SATurated fat and gene *APOE* (SATgenε) study [15] prospectively recruited males and females based on their *APOLIPOPROTEIN (APO)E* genotype (*APOE3/E3* or *APOE3/E4*). The observational study Impact of physiological and lifestyle factors on BODY COmpositioN (BODYCON) included disease-free adults aged between 18-70 years. Since these studies used similar inclusion/exclusion criteria and standardised measurements of dietary intake, anthropometric measures and CMD risk markers, baseline data could be pooled to create a larger cross-sectional dataset.

We followed the Strengthening the Reporting of Observational Studies in Epidemiology-Nutritional Epidemiology (STROBE-nut) guidelines to report our findings [20].

### Demographics, anthropometric and biochemical measurements

For all studies, participants attended the Hugh Sinclair Unit of Human Nutrition, following an overnight fast and consumption of a standard low fat evening meal, with limits imposed on the level of physical activity and alcohol consumption in the 24-h period preceding the scheduled study visit. ​Participants completed questionnaires to self-report demographics, including sex, age, menopausal status and ethnicity (information on ethnicity was not captured in the SATgenε study) and health status. A standardised technique for performing anthropometric measurements and blood pressure was used in all studies, as documented in previous publications [15–19, 21, 22]. Briefly, height (cm, stadiometer), weight (kg, Tanita BC-418, TANITA UK Ltd in Middlesex, UK), and waist circumference (WC) (cm, non-stretch tape measure, (Seca, UK)) were measured. Body mass index (BMI) (kg/m^2^) was calculated. All clinic blood pressure measurements were taken in triplicate from the upper arm whilst the participants were at rest using an Omron digital automatic upper arm blood pressure monitor (Omron Healthcare Co UK Ltd), and the mean systolic blood pressure (SBP) and diastolic blood pressure (DBP) measurements were calculated. Pulse pressure (PP) was determined by subtracting the mean DBP from the mean SBP.

The ILAB 600 (Werfen (UK) Ltd, Warrington, UK) and the Daytona Plus (RANDOX Laboratories Ltd, Crumlin, UK) clinical chemistry analysers were used to quantify fasting lipids including total cholesterol (TC), high-density lipoprotein-cholesterol (HDL-C) and triacylglycerol (TAG), as well as fasting glucose using kits supplied by the manufacturers of the equipment. The Friedewald formula [23] was used to calculate the fasting low-density lipoprotein-cholesterol (LDL-C) concentration. In addition, non-HDL-C was calculated by subtracting HDL-C from TC, and remnant-like particle-cholesterol (RLP-C) by subtracting LDL-C from non-HDL-C. The TC:HDL-C ratio was calculated by dividing the TC by the HDL-C concentration. Serum insulin concentrations in the DIVAS, DIVAS-2, RESET and SATgenɛ studies were analysed using an ELISA kit obtained from Dako Ltd (High Wycombe, UK) whereas in the BODYCON study, a Simple Plex insulin assay and the automated ELISA platform Ella (Bio-Techne) was used. The homeostatic model assessment estimated insulin resistance (HOMA-IR) was also calculated [24].

### Dietary assessment and food categorisation

Details on the dietary assessment techniques used in the different studies have been previously published [15–19, 21, 22]. In brief, participants’ habitual food, drink and dietary supplement intakes were recorded using weighed diet diaries over 3 days (SATgenε) or 4 days (DIVAS, DIVAS-2, BODYCON, RESET) within a 7-day period and each study included one weekend day [25]. After being checked for completeness (where necessary, additional details were requested from participants), they were analysed using the Dietplan dietary analysis software (Forestfield software: version 7, except DIVAS that used version 6.6), where the food and macro- and micro-nutrient intake data (excluding dietary supplements) were generated for each study. Mean daily nutrient intakes were calculated by dividing the total intakes by number of days recorded.

To pool the datasets, food-level datasets were exported from Dietplan (one dataset per study), which included g/d and nutrient composition of each food item consumed per participant. Using these datasets, each food/drink item a participant consumed during the recording period was matched (by AY) to one of 137 pre-defined food categories, defined by study researchers (MW and JAL), that represented foods and drinks typically consumed in the UK diet. Use of dietary supplements was also categorised (yes/no). Each categorised dataset was then independently reviewed by a second researcher to identify any misclassified items. For each participant, g/d of multiple food items consumed within a single food category (e.g., “berries” consisted of strawberries, raspberries, blueberries, etc.) were summed, and the total intakes were divided by the number of days recorded to give the mean daily intake of each food category per participant. Categorised data (g/d) for all participants from each study were then merged to create a single dataset. Given the nature of diet diary recording, participants recorded food/drink items as either unprepared weights (e.g., raw, dried, concentrated, with wastage included such as bones) or weights as consumed (e.g., cooked, rehydrated, diluted, without wastage included), and foods (e.g. meat, fish, pasta, rice) were categorised accordingly. All food categories recorded as unprepared weights were converted to consumed weight equivalents using standard conversion formulas (e.g., % weight changes and edible factors) [26] or conversion factors from other sources, such as the UK Department of Health’s Sampling Reports [27] and food label information on supermarket websites.

Many of the 137 food categories were merged with similar items, which resulted in 38 aggregated food categories for use in PCA to ease the extraction of the components as outlined in Supplementary Table 2. Decisions for including these 38 groupings were based on evidence regarding their relationship with CMD risk. For example, a recent systematic review [28] suggested the type of dairy product (e.g., cheese, yogurt, or milk), rather than the fat content of the product, had more influence on CVD risk, so dairy products were classified as "milk" and "fermented dairy (cheese and yoghurt)". Soft drinks, another example, were split into “sweetened beverages” and “low/free-sugar beverages" due to the observed association between sugar-sweetened and artificially-sweetened beverages and obesity [29].

To facilitate calculation of the AHEI-2010 diet quality score, food group intakes (such as total fruits, vegetables, wholegrain products, and processed meats) were calculated. These values also included contributions from composite food items, which were based on a dataset created in-house that disaggregated composite food items into their component parts, similar to the approach used by the National Diet and Nutrition Survey [30]. For each composite food category (e.g. pizza), a range of food items (e.g. meat pizza, cheese pizza) typically consumed in the UK were deconstructed using ingredient information on food labels from UK supermarkets. This was repeated for multiple brands per food item, from which the average proportions of cheese, vegetables, processed meat, etc. were calculated. In addition, two nutrients not generated by Dietplan were estimated using the categorised data. Long chain n-3 polyunsaturated fatty acids (PUFA) were estimated from participants’ intakes of white fish, oily fish and other seafood plus the % of these found within composite food categories (such as fish-based ready meals, sushi and breaded fish products). Intakes of free sugars (g/d) were also estimated based on the UK’s definition of free sugars [31] in which i) total sugars (g/d) from fruits and vegetables [including whole, canned/stewed (estimated 77% fruit content) and dried types, but not blended types, e.g., juices, smoothies, sauces and soups] and ii) total lactose (g/d) were excluded from the participants’ daily total sugar intake (g/d).

### AHEI-2010 calculation

The AHEI-2010 was chosen to assess diet quality since this diet quality score evaluates adherence to intakes of 11 foods and nutrients which are predictive of chronic disease risk [32]. These components include vegetables, fruits, wholegrains, nuts and legumes, long chain n-3 PUFA, total PUFA, sugar-sweetened beverages, red and processed meats, trans fats, sodium, and alcohol. Each component receives a score from 0 to 10 points, with higher scores reflecting greater alignment with dietary recommendations. The total score is out of 110 points and higher scores reflect greater diet quality [32].

### Statistical analysis

For this cross-sectional analysis, at least 3 days of diet diary recording and feasible energy intakes between 500 and 3500 kcal/d for females and 800 and 4200 kcal/d for males were required for dietary data inclusion. Individuals with energy intakes outside of these ranges were previously considered to be under and over reporters [33]. Those without any outcome data (anthropometric and biochemical measures) were also excluded. In addition, if participants took part in multiple studies, only one dataset was retained for statistical analysis based on the following hierarchy: DIVAS > DIVAS-2 > RESET > BODYCON > SATgenɛ.

PCA with orthogonal (varimax) rotation was applied in our cohort to extract unrelated components (i.e., main DPs). Suitability of the data for PCA was assessed using the Kaiser–Meyer–Olkin (KMO) value (> 0.6 to indicate sampling adequacy) and Bartlett's test of sphericity (*p* < 0.05 to indicate suitability for data reduction) [34]. Food groups (*n* = 38, expressed as mean g/d) were included in the PCA analysis and those with factor loadings of ≥ 0.3 or ≤ 0.3 were considered important contributors to the DP in line with previous studies [11, 35]. The selection of DPs (principal components) was based on eigenvalues ≥ 1.5, which have been used in other nutrition studies to identify the smallest number of patterns explaining the largest variance in food intake [11] followed by a visual assessment of the scree plot (Supplementary Figure 1). Participant factor scores were then classified into quartiles for each DP [Quartile (Q) 1: lowest adherence to Q4: highest adherence] to determine the associations with CMD risk markers, nutrient intakes and diet quality.

Prior to the analysis of covariance (ANCOVA), normality was assessed for dependent variables using the Kolmogorov–Smirnov test, histograms, and Q-Q plots. Serum TC, LDL-C, and non-HDL-C were normally distributed variables, while all others were skewed and normalized using a log10 transformation. Due to the high proportion of non-consumers of alcohol, a constant value of 1 was added to the alcohol intake data prior to transformation. ANCOVA was used to assess variations in estimated marginal means of anthropometric measurements, CMD risk biomarkers, AHEI-2010 score, and daily nutrient intakes between quartiles of DP adherence. Factors widely considered to affect CMD risk and dietary intake were pre-selected as covariates, including: age (years), sex (male or female), consumption of dietary supplements (yes or no), menopausal status [classified as pre, peri, post, not specified or n/a (male)], energy intake (kcal/d), and whether the study population was recruited based on moderate CVD risk (yes or no). As macronutrients were expressed as a percentage of total energy (%TE) and had already been adjusted for energy, daily energy intake was not included as a covariate for these analyses. In the case of a significant result in the fully adjusted model, Bonferroni correction was automatically applied to the pairwise comparisons to account for multiple testing. P-trend was also calculated within the ANCOVA as a secondary statistical analysis. In this study, a more conservative significance level of *P* ≤ 0.01 was used for all statistical tests. All analyses were conducted using IBM SPSS Statistics Version 27 (IBM Corp.).

## Results

**Fig. 1:**
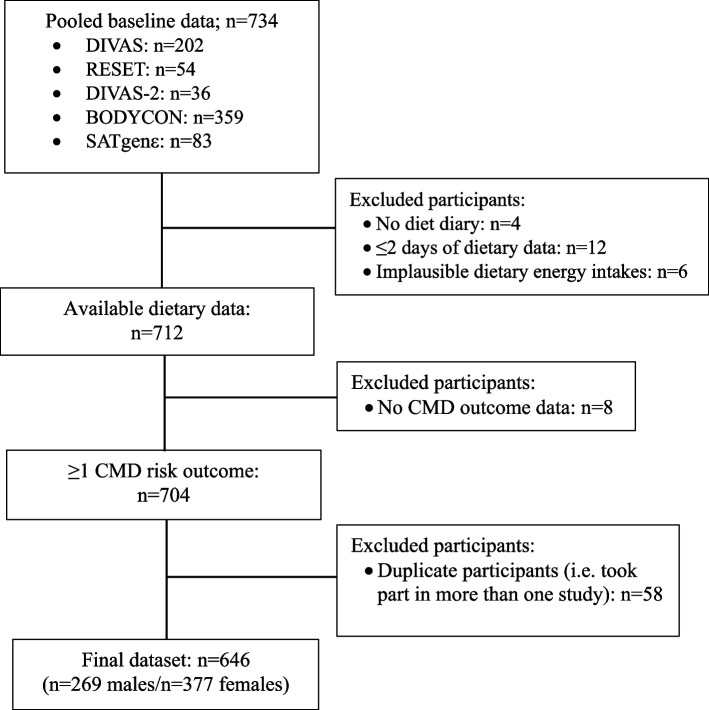
Flowchart of study selection and inclusion for pooled dataset. *Abbreviations*: BODYCON, impact of physiological and lifestyle factors on body composition; CMD, cardiometabolic disease; DIVAS, dietary intervention and vascular function, RESET, replacement of saturated fat in dairy on total cholesterol, SATgenε, SATurated fat and gene *APOE*

Of the 734 individuals included in the five human studies, six adults were excluded due to implausible dietary energy intakes, 16 were excluded with ≤ 2 days of dietary data, 8 due to missing outcome data and 58 adults participated in multiple human studies (Fig. 1). A total of 646 (58.4% female) self-reported disease-free adults were included in this cross-sectional data analysis. The mean (SD) age of the cohort was 44 (14) years, BMI was 25.2 (4.0) kg/m^2^ (44% overweight and 13% obese), and the mean AHEI-2010 score was 59 (15) out of 110. The participants self-identified their ethnic group as White (75.5%), Asian or Asian British (9.8%), Black, Black British, Caribbean or African (2.8%), Mixed (1.1%), Other (0.6%) or not recorded (10.2%).

### Dietary patterns analysis

**Fig. 2:**
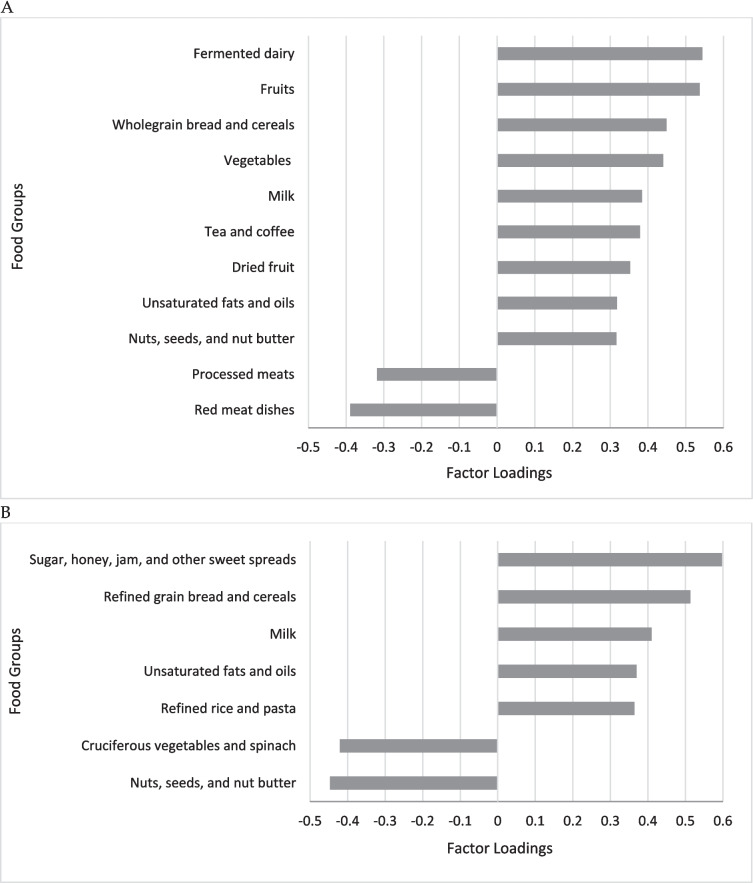
Factor loadings for (**A**) dietary pattern 1, (**B**) dietary pattern 2 in disease-free UK adults identified by PCA. Factor loadings ≥0.3 and ≤−0.3 were used to identify food groups that characterised the dietary patterns.

After confirming the data were appropriate for PCA (KMO = 0.641; Bartlett’s test of sphericity = *p* < 0.001), five DP were identified as having eigenvalues of > 1.5, explaining the greatest proportions of variance in our food consumption dataset. Two DPs were then retained following a visual exploration of the scree plot (Supplementary Figure 1), which accounted for the largest variation (approximately 12%: 6.0% for DP1 and 5.7% for DP2). Rotated factor loadings for all 38 food groups for DP1 and DP2 are presented in Supplementary Table 2. Food group characteristics of each DP are shown in Fig. 2. DP1 was characterised by higher intakes of fermented dairy, fruits (including dried fruits), wholegrains, vegetables, milk, tea and coffee, nuts/seeds, and unsaturated fats/oils and lower intakes of red meat dishes and processed meats. DP2 was characterised by higher intakes of sugar and sweet spreads, refined grains, and unsaturated fats/oils, and lower intakes of cruciferous vegetables/spinach and nuts/seeds (Supplementary Table 2).

Due to the similarity of *p*-values for the p-trend and ANCOVA for the associations between DP and CMD risk markers and nutrient intakes, only the findings for the ANCOVA (primary analysis) will be presented in the paper.

### Associations between the dietary patterns and CMD risk markers

CMD risk factors are presented according to quartiles of adherence to each DP in Table 1. Those in the highest quartile (Q4) of DP1 had a significantly lower BMI and WC compared to those in Q1 and Q2 (*P* ≤ 0.003) (Table 1), whereas those in Q3 and Q4 had lower DBP than those in Q1 (*P* ≤ 0.007). Relative to Q1, fasting TAG and RLP-C were lower whereas HDL-C was higher in Q4 (*P* ≤ 0.003). For non-HDL-C, concentrations were lower in Q4 compared with Q1 and Q2 (*P* ≤ 0.005). TC:HDL-C ratio was lower in Q3 and Q4 also differing to Q2 (*P* ≤ 0.004). There was no association between DP1 and SBP, PP, fasting TC concentration or measures of glycaemic control (including fasting glucose, insulin and HOMA-IR). Although the ANCOVA overall effect for serum LDL-C was significant for DP1, post-hoc comparisons between quartiles did not reach the level of statistical significance.

**Table 1:** Cardiometabolic disease risk markers in disease-free UK adults according to quartiles of adherence to dietary patterns 1 and 2^1^

*Risk factors, units*	n	Dietary Pattern 1					Dietary Pattern 2				
		Q1 (*n*=162)	Q2 (*n*=161)	Q3 (*n*=161)	Q4 (*n*=162)	P*	Q1 (*n*=162)	Q2 (*n*=160)	Q3 (*n*=162)	Q4 (*n*=162)	P*
Anthropometric measures and blood pressure											
BMI, *kg/m*^*2*^^§^	646	26.0 (0.3)^a^	25.6 (0.3)^a^	24.9 (0.3)^ab^	24.0 (0.3)^b^	<0.001	25.3 (0.3)	25.2 (0.3)	24.8 (0.3)	25.3 (0.3)	0.643
WC, *cm*^§^	644	88.9 (0.8)^a^	87.6 (0.8)^a^	85.7 (0.8)^ab^	83.0 (0.8)^b^	<0.001	86.4 (0.8)	86.3 (0.8)	85.4 (0.8)	87.1 (0.8)	0.606
SBP, *mmHg*^§^	645	124 (1)	121 (1)	120 (1)	119 (1)	0.021	120 (1)	121 (1)	122 (1)	120 (1)	0.716
DBP, *mmHg*^§^	645	75 (1)^a^	73 (1)^ab^	72 (1)^b^	71 (1)^b^	0.002	72 (1)	73 (1)	73 (1)	72 (1)	0.395
PP, *mmHg*^§^	645	49 (1)	47 (1)	48 (1)	48 (1)	0.343	48 (1)	48 (1)	49 (1)	48 (1)	0.864
Biochemical risk markers											
TC, *mmol/l*	642	5.43 (0.08)	5.43 (0.08)	5.23 (0.08)	5.15 (0.08)	0.027	5.34 (0.08)	5.37 (0.08)	5.27 (0.08)	5.26 (0.08)	0.734
TAG, *mmol/l*^§^	642	1.24 (0.04)^a^	1.11 (0.04)^ab^	1.05 (0.04)^ab^	0.99 (0.04)^b^	0.003	1.06 (0.04)	1.07 (0.04)	1.10 (0.04)	1.16 (0.04)	0.228
LDL-C, *mmol/l*^§^	642	3.37 (0.07)^a^	3.35 (0.07)^a^	3.16 (0.07)^a^	3.05 (0.07)^a^	0.003^†^	3.21 (0.07)	3.30 (0.07)	3.19 (0.07)	3.24 (0.07)	0.657
HDL-C, *mmol/l*^§^	642	1.50 (0.03)^a^	1.57 (0.03)^ab^	1.59 (0.03)^ab^	1.65 (0.03)^b^	<0.001	1.65 (0.03)^a^	1.58 (0.03)^ab^	1.59 (0.03)^ab^	1.50 (0.03)^b^	0.006
TC:HDL-C ratio^§^	642	3.85 (0.08)^a^	3.62 (0.07)^ab^	3.42 (0.07)^bc^	3.22 (0.08)^c^	<0.001	3.44 (0.08)	3.54 (0.07)	3.47 (0.07)	3.67 (0.08)	0.053
Non-HDL-C, *mmol/l *	642	3.94 (0.08)^a^	3.86 (0.07)^a^	3.63 (0.07)^ab^	3.50 (0.08)^b^	<0.001	3.69 (0.08)	3.79 (0.07)	3.68 (0.07)	3.77 (0.08)	0.718
RLP-C, *mmol/l*^§^	642	0.56 (0.02)^a^	0.51 (0.02)^ab^	0.48 (0.02)^ab^	0.45 (0.02)^b^	0.003	0.48 (0.02)	0.49 (0.02)	0.50 (0.02)	0.53 (0.02)	0.228
Glucose, *mmol/l*^§^	642	5.16 (0.04)	5.09 (0.04)	5.06 (0.04)	5.04 (0.04)	0.107	5.08 0.04)	5.07 (0.04)	5.08 (0.04)	5.12 (0.04)	0.806
Insulin, *pmol/l*^§^	635	31.2 (1.4)	30.4 (1.4)	31.9 (1.4)	29.2 (1.4)	0.774	30.8 (1.5)	29.0 (1.4)	30.0 (1.4)	33.0 (1.5)	0.411
HOMA-IR^§^	635	1.19 (0.06)	1.17 (0.06)	1.22 (0.06)	1.12 (0.06)	0.768	1.18 (0.06)	1.09 (0.06)	1.15 (0.06)	1.26 (0.06)	0.271

*Abbreviations* *BMI* body mass index, *DBP* diastolic blood pressure, *DP* dietary pattern, *HDL-C* high-density lipoprotein-cholesterol, *HOMA-IR* homeostatic model assessment estimated insulin resistance, *LDL-C* low-density lipoprotein-cholesterol, *PP* pulse pressure, *RLP-C* remnant-like particle-cholesterol, *SBP* systolic blood pressure, *TAG* triacylglycerol, *TC* total cholesterol, *WC* waist circumference

^1^Data represents the estimated marginal means (standard error) stratified according to the factor loadings for each DP. The factor loadings were as follows: DP1 Q1 (−2.822 to −0.651), Q2 (−0.649 to −0.070), Q3 (−0.067 to 0.546), and Q4 (0.553 to 4.621) and DP2 Q1 (−4.621 to −0.540), Q2 (−0.539 to 0.007), Q3 (0.010 to 0.557), and Q4 (0.563 to 4.269)

^§^Log10 transformed prior to statistical analysis. Estimated marginal means are shown for the untransformed data

^*^p≤0.01 was considered significant for ANCOVA after adjusting for covariates (sex, age, menopausal status, supplement usage, energy intake, and cardiovascular disease risk) and pairwise comparisons with Bonferroni correction. Different superscript letters

^a,b,c^ represent significant differences between quartile groups

Of the CMD risk markers measured, only HDL-C was found to be different across increasing quartiles of DP2, with Q4 associated with a lower concentration compared with Q1 (*P* = 0.004).

### Associations between the dietary patterns with nutrient intake and diet quality

Nutrient intakes and AHEI-2010 scores are presented according to quartiles of adherence to each DP in Supplementary Table 3. In DP1, individuals in Q4 had the highest intakes of n-6 PUFA and carbohydrate (*P* = 0.001), and lowest intakes of sodium (*P* < 0.001) and free sugars (*P* = 0.01) compared to Q1. There was a significant stepwise increase in fibre intakes from Q1 to Q4 (all *P* < 0.001), which was similar for total sugars although Q2 and Q3 were not significantly different. For SFA, intakes in Q4 were significantly lower than Q1 and Q2 (*P* = 0.002), whereas intakes of alcohol in Q3 and Q4 were lower than Q1 (*P* ≤ 0.006). There was a significant stepwise increase in AHEI-2010 scores from Q1 to Q4 (all *P* < 0.001).

For DP2, individuals in Q4 had the lowest total fat intakes compared to Q1 and Q2 (*P* ≤ 0.006), with intakes also lower in Q3 versus Q1 (*P* < 0.001). Relative to Q1, MUFA and n-6 PUFA intakes were lower in Q3 and Q4 (*P* ≤ 0.002), whereas protein intakes were higher in Q1 versus the other quartile groups (all *P* ≤ 0.006). Q1 had lower free sugar intakes compared with Q4 (*P* < 0.001), whereas total carbohydrate intakes were significantly higher in Q2, Q3 and Q4 versus Q1 (all *P* < 0.001) and in Q4 compared with Q2 (*P* < 0.001). Relative to Q1 and Q2, AHEI-2010 scores were significantly lower in Q3 and Q4 (*P* < 0.001).

## Discussion

In the current cross-sectional analysis, two distinct DPs were identified in self-reported disease-free adults residing in and around the Reading area, UK. DP1 was broadly aligned with population dietary guidelines (EatWell Guide), with higher intakes of fermented dairy, fruits, vegetables, wholegrains, nuts/seeds, milk and unsaturated fats/oils and lower intakes of red meat dishes and processed meat. In contrast DP2 was a mixed, less healthful DP that was less compliant with UK dietary guidelines characterised by higher intakes of foods groups considered less healthy, including free sugars and refined grains (refined carbohydrates) and healthier food groups (unsaturated fats/oils, and milk) as well as lower intakes of cruciferous vegetables/spinach, and nuts/seeds. In line with our hypothesis, participants with the greatest adherence to a more healthful DP1, which broadly aligns with UK dietary guidelines, had higher diet quality and a more favourable CMD risk factor profile.

Increasing adherence to DP1 was related to more favourable anthropometric measures (BMI, WC), DBP and fasting lipid profile (TAG, HDL-C, TC:HDL-C ratio), whereas greater adherence to DP2 had little association with CMD risk, apart from lower HDL-C. Other studies using data-driven a posteriori approaches have shown similar relationships with more healthful/prudent DP, particularly with BMI and WC, but relationships with the lipid profile, blood pressure and markers of glycaemic control were inconsistent. In agreement with our findings, both BMI and prevalence of overweight and obesity were lower for Austrian adults following a ‘health conscious’ DP compared with those following a ‘Western’ or ‘traditional’ DP [36]. Likewise, a 'health aware' DP was positively associated with HDL-C in middle-aged UK adults [7]. Furthermore, deciles of adherence to a ‘healthy’ DP were inversely related to TAG and BMI in American Indians, although, unlike our findings, this study also reported negative associations for LDL-C, HDL-C, SBP and HOMA-IR [37]. Similarly, adherence to a DP rich in fruits, vegetables, wholegrains, poultry and legumes in middle-aged Iranian women was linked to lower serum TAG, BMI, WC, blood pressure, and odds of being insulin resistant and higher HDL-C. However, this DP was also associated with higher physical activity levels and lower energy intake, which could have contributed to these findings [38]. Furthermore, a ‘prudent’ DP was negatively associated with WC, TAG, insulin and frequency of the metabolic syndrome and positively associated with QUICKI (insulin sensitivity) in young US adults, with a lack of relationship for TC, LDL-C, HDL-C, blood pressure, glucose and HOMA-IR [39]. Interestingly, after further adjustment for BMI, only WC and insulin sensitivity remained significant. Finally, different DPs were identified in UK males and females, with only a healthful DP rich in fruit, vegetables and dairy products found in females. This DP was inversely associated with BMI, WC, and blood pressure, but not associated with the blood lipid profile [5].

In our data analysis, higher adherence to the mixed, less healthful DP (DP2) was related to a 9% lower HDL-C, on average. The UK Whitehall II study [6] of middle-aged civil servants (aged 35-55 years) also identified a DP partially characterised by higher consumption of sugar in tea and coffee and white bread (refined grains) and lower intakes of vegetables [6]. Consistent with our findings, higher adherence to this DP was associated with a lower HDL-C, although it was also correlated with higher TC and TAG in the Whitehall II study. Prospective analysis also revealed higher adherence to this DP was related to an increased coronary heart disease risk over a 15-year follow-up, but this relationship was attenuated after further adjustment for blood pressure and BMI. In the UK Biobank, the DP which explained the highest variation in dietary data was defined as an unhealthy diet score. In line with our findings, higher adherence to this DP was associated with a lower HDL-C but this study also found associations with a less favourable lipid profile (higher TC, apoB and TAG), higher glycated haemoglobin, C-reactive protein, BMI and blood pressure [13]. Consumption of sugar and refined carbohydrates, characteristic of DP2, have been inversely associated with HDL-C in both randomised controlled trials and longitudinal studies [40, 41], with glycaemic index and glycaemic load considered as important determinants of the relationship between carbohydrate intake and HDL-C. Therefore, higher free sugar intakes with greater adherence to DP2 in the current study may have contributed in part to the lower HDL-C. Anecdotally, breads and breakfast cereals are typically consumed with fat spreads and milk, respectively, in the UK, which may somewhat explain the combination of both healthier and less healthy food groups with positive factor loadings in DP2. Although this mixed DP was considered less healthful than DP1, it did not fully resemble a typical Western DP. Whereas high intakes of refined carbohydrates with low intakes of vegetables, and nuts/seeds were characteristic of both DP2 and the Western DP, the latter is also typically characterised by high intakes of red and processed meats, fried foods and sugar-sweetened drinks, and low intakes of fruits [42], which were not apparent in DP2. Since DP2 was also associated with higher intakes of more nutrient-dense foods (milk and unsaturated fats/oils), this may partially explain the lack of unfavourable associations between greater adherence to DP2 and CMD risk factors, which are often observed with the traditional Western DP [43–45].

Specific foods, nutrients and bioactive compounds within the DPs may have contributed to the findings. The food groups characteristic of DP1, including higher intakes of fruits, vegetables, wholegrains and dairy, are key aspects of the evidence-based Dietary Approaches to Stop Hypertension DP [46], which is often promoted to lower blood pressure and reduce CVD risk. Although small, the 4 mmHg lower DBP observed in Q4 vs Q1 of DP1 may be clinically relevant given that a 5 mmHg increase in DBP has previously been associated with a 4% higher risk of cardiovascular events [47]. This may be partially attributed to higher intakes of fruit, vegetables, wholegrains and dairy, which have been shown in systematic reviews and meta-analyses to be inversely associated with risk of hypertension and raised blood pressure [48–50]. Moreover, dietary intakes of sodium, which are often characteristic of processed meats, has well-evidenced hypertensive effects [51], with intakes found to be lower with increasing adherence to DP1.

There is current evidence that higher intakes of dairy foods, notably fermented products, are cardioprotective [52, 53], with our study showing Q4 to consume on average 4-times as much fermented dairy (cheese and yogurt) compared with the lowest adherence to DP1 (109 g/d vs 26 g/d). Findings from randomised controlled trials have shown fermented milk products to have antihypertensive effects [54], where bioactive peptides generated during fermentation are purported to mediate the beneficial effects on vascular function [55]. Similar to our findings, the Norwegian Tromsø Study reported an inverse association between total fermented dairy intake and TAG in adults, but not with TC or LDL-C. Although we found a positive association with HDL-C, the latter study reported no association even though intakes of yogurt and cheese were similar between studies. However, this inconsistency may be attributed to different food sources being classified as fermented dairy in each study (our study classified yogurt and cheese as fermented dairy, whereas the Norwegian study included yogurt, cheese, sour cream, and cultured milk/kefir) [56]. A systematic review of cohort studies reported the type of dairy was more important than fat content in relation to CVD risk, in which fermented dairy products were associated with a lower risk, whereas milk, full fat dairy and low-fat dairy all had neutral effects [28]. Since many of the healthful/prudent DPs identified above were characterised by higher intakes of low-fat dairy, differences in dairy product categorisation may have impacted the relationships found with CMD risk markers.

The findings from our study suggest that following a diet with the highest (Q4) vs lowest (Q1) adherence to DP1 could be associated with clinically significant benefits in BMI (a difference of −2 kg/m^2^ reflecting normal weight vs overweight BMI categories, respectively), blood pressure (−4 mmHg DBP) and serum lipids (e.g. + 0.15 mmol/l HDL-C), as indicated in the literature [47, 57, 58]. These favourable associations in our study population of self-reported disease-free adults reinforces current UK food-based recommendations to consume a diet higher in fruits, vegetables, wholegrains, nuts/seeds, dairy and unsaturated fats/oils and lower in red and processed meats. Although this DP aligns well with the Eatwell Guide, there are some differences. For example, the guide recommends UK adults consume some milk and dairy foods, such as cheese and yogurt, without a distinction between fermented and non-fermented dairy. In view of the growing evidence base for the importance of the type of dairy foods on health outcomes, our findings would support food-based recommendations that emphasised the consumption of both fermented and non-fermented dairy foods rather than relying solely on milk consumption for CMD prevention.

There are several strengths of this study. Weighed diet diaries were used to assess dietary intake, which provide good estimates of nutrient and food group intakes [14]. Data from five studies conducted within a single centre employing standardised methodologies, equipment and laboratory techniques were combined to generate the dataset of 646 adults. Although considered small for an observational study, our sample size is comparable to other published studies using data-driven approaches to identify DP [59]. Another limitation was the cross-sectional design which cannot prove cause and effect. Our dataset included self-reported disease-free adults living in and around the Reading area (South East of England) with moderate diet quality and a low-to-moderate risk of CVD so we acknowledge that our findings may not necessarily be transferable to the general UK population as a whole. Although heterogeneity in the studied populations could impact on the results, these factors (such as age, sex and menopausal status) were considered in the analysis as covariates. In addition, data were pooled from studies conducted over a 10-year period and as such food groups and nutrient intakes may not be representative of current diets. Furthermore, it must be acknowledged that using a data-driven approach, derived DPs are study specific and relevant to many factors, such as subject group, classification and number of food groups, and statistical approach (such as eigenvalues and factor loadings) [2], which inherently restricts the ability to directly compare findings between studies.

In conclusion, this analysis identified two common DPs in UK adults. Greater adherence to a DP broadly aligned with UK dietary guidelines (DP1) was associated with a favourable CMD risk marker profile and a higher diet quality, whereas greater adherence to a mixed, less healthful DP (DP2) was related to lower HDL-C and diet quality. Our findings could be used to inform future UK food-based dietary recommendations, particularly highlighting the importance of fermented dairy foods.

## Supplementary Information

Supplementary Material 1.

## Data Availability

The data that support the findings of this study are available from the corresponding author upon reasonable request.

## Abbreviations

AHEI-2010: Alternative Healthy Eating Index-2010
ANCOVA: Analysis of Covariance
*APOE*: *APOLIPOPROTEIN (APO)E*
BMI: Body mass index
BODYCON: Impact of physiological and lifestyle factors on BODY COmpositioN
CMD: Cardiometabolic disease
CVD: Cardiovascular disease
DBP: Diastolic blood pressure
DIVAS: Dietary Intervention and VAScular function
DP: Dietary pattern
HDL-C: High-density lipoprotein-cholesterol
HOMA-IR: Homeostatic model assessment estimated insulin resistance
KMO: Kaiser–Meyer–Olkin
MUFA: Monounsaturated fatty acids
LDL-C: Low-density lipoprotein-cholesterol
PCA: Principal component analysis
PUFA: Polyunsaturated fatty acids
PP: Pulse pressure
QUICKI: Quantitative insulin sensitivity check index
RLP-C: Remnant-like particle-cholesterol
RESET: REplacement of SaturatEd fat in dairy on Total cholesterol
SATgenε: SATurated fat and gene *APOE*
SBP: Systolic blood pressure
SFA: Saturated fatty acids
TAG: Triacylglycerol
TC: Total cholesterol
WC: Waist circumference
