# Supplementary material for: Association between empirically driven dietary patterns and cardiometabolic disease risk factors: a cross-sectional analysis in disease-free adults
Source: Nutr Metab (Lond). 2025 Jul 9;22:73. doi: 10.1186/s12986-025-00965-6 (PMC12239307; doi:10.1186/s12986-025-00965-6)
Supplement: Supplementary file 1 — Supplementary Material 1. [file 12986_2025_965_MOESM1_ESM.docx]

**Supplementary Materials**

**Supplementary Table 1.** Summary of the study participants included in the baseline data analysis.

| **Study name** | **Sample size (n)** | **Population recruited** | **Recruitment dates** | **ClinicalTrials.gov registration numbers** |
| --- | --- | --- | --- | --- |
| SATgenε | 88 | Males and females aged 35-70 y, *APOE3/E3* and *APOE4* carrier groups matched for age, sex, and BMI | 2009-2011 | NCT01384032 |
| DIVAS | 202 | Males and females aged 21-69 y with moderate CVD risk^1^ | 2009-2012 | NCT01478958 |
| DIVAS-2 | 36 | Females aged 48-65 y, post-menopausal | 2014-2015 | NCT02144454 |
| RESET | 54 | Males and females aged 25-70 y with moderate CVD risk^1^ | 2014-2016 | NCT02089035 |
| BODYCON | 409 | Males and females aged 18-70 y | 2015-2019 | NCT02658539 |

**^1^**To identify individuals with moderate CVD risk, a scoring tool based on the Framingham risk prediction algorithm was used at screening [7,10].

Abbreviations: BODYCON, impact of physiological and lifestyle factors on BODY COmpositioN; DIVAS, Dietary Intervention and VAScular function; RESET, REplacement of SaturatEd fat in dairy on Total cholesterol; SATgenε, SATurated fat and gene *APOE*

KMO:0.641, P<0.001


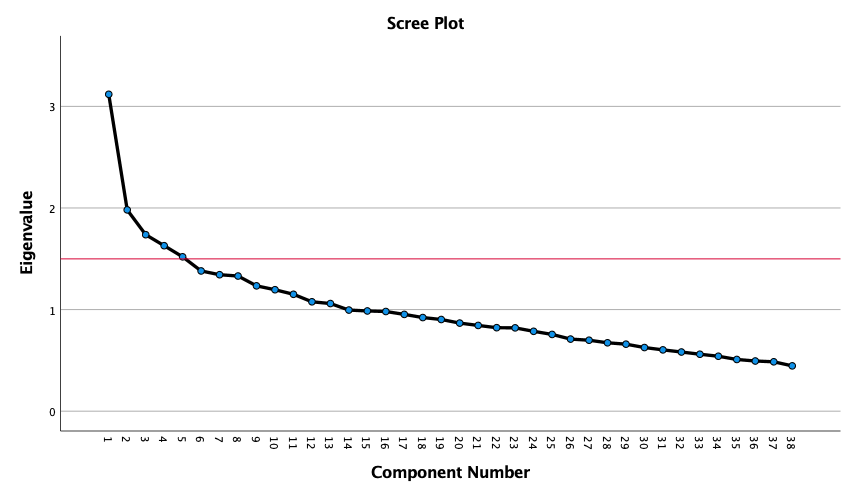


1 2 3 4 5 6 7 8 9 10 11 12 13 14 15 16 17 18 19 20 21 22 33 24 25 26 27 28 29 30 31 32 33 34 35 36 37 38

**Supplementary Figure 1.** Scree plot of the eigenvalues for each of the 38 food category components, after applying the principal component analysis orthogonal varimax rotation method. Eigenvalues >1.5 were the cut-off point of selection to identify the smallest number of dietary patterns explaining the largest variance in food intake (highlighted by red line). Abbreviations: KMO, Kaiser-Meyer-Olkin.

| **Food Groups**  **Supplementary Table 2.** Rotated component loadings derived from principal component analysis for dietary variables that characterise the 2 main dietary patterns identified. | **Rotated component factor loadings^1^** | | |
| --- | --- | --- | --- |
|  | **Dietary Pattern-1** | | **Dietary Pattern-2** |
| Fermented dairy (cheese and yogurt) | | **0.544** | -0.040 |
| Milk (any dairy type) | | **0.384** | **0.410** |
| Dairy alternative products | | 0.099 | -0.292 |
| Wholegrain bread and cereals | | **0.449** | 0.032 |
| Wholegrain rice and pasta | | 0.209 | -0.260 |
| Refined bread and cereals | | -0.110 | **0.513** |
| Refined rice and pasta | | -0.136 | **0.364** |
| Nuts, seeds and nut butter | | **0.316** | **-0.447** |
| Fruits (including whole, canned and stewed) | | **0.537** | -0.171 |
| Dried fruit | | **0.353** | -0.112 |
| Vegetables (excluding peas/beans, cruciferous vegetables and spinach) | | **0.440** | -0.295 |
| Cruciferous vegetables (e.g., broccoli, cabbage) and spinach | | 0.204 | **-0.421** |
| Vegetable dishes | | -0.088 | 0.036 |
| Potatoes and potato products/dishes | | 0.004 | 0.194 |
| Legumes (e.g., green beans, chickpeas, lentils) and legume dishes | | 0.183 | 0.023 |
| Meat alternative products and dishes | | 0.044 | -0.089 |
| Pizza | | -0.241 | -0.068 |
| Red meat (unprocessed) | | -0.167 | 0.076 |
| Poultry (unprocessed) | | 0.112 | -0.283 |
| Processed meat | | **-0.318** | -0.002 |
| Red meat dishes | | **-0.389** | 0.015 |
| Poultry dishes | | -0.069 | -0.040 |
| Eggs and egg dishes | | -0.057 | -0.262 |
| Oily fish (unprocessed) | | 0.061 | -0.159 |
| White fish, tuna, and shellfish (unprocessed) | | 0.023 | 0.091 |
| Fish products and dishes | | 0.118 | 0.028 |
| Unsaturated fats and oils (e.g., olive oil, sunflower oil spread) | | **0.318** | **0.370** |
| Saturated fats and oils (e.g., butter, lard, coconut oil) | | -0.006 | 0.086 |
| Condiments, table sauces, and savoury spreads | | 0.113 | 0.064 |
| Sugar, honey, jam, and other sweet spreads | | 0.111 | **0.597** |
| Cream and dairy-based desserts | | 0.033 | -0.043 |
| Snacks (e.g., cakes, biscuits, confectionery and potato crisps) | | -0.038 | 0.172 |
| Alcoholic drinks | | -0.188 | 0.036 |
| Tea and coffee (excluding added milk and sugar) | | **0.379** | 0.219 |
| Pure fruit and vegetable juices and smoothies | | 0.095 | 0.192 |
| Low-sugar and sugar-free beverages | | 0.013 | -0.280 |
| Sugar-sweetened beverages | | -0.202 | 0.052 |
| Variance of food intake explained (%) | | 6.0 | 5.7 |

^1^Bold numbers indicate food groups with absolute factor loadings >0.3 and <0.3 which characterised each dietary pattern.

**Supplementary Table 3.** Selected nutrient intake and AHEI-2010 according to quartiles of adherence to dietary patterns 1 and 2^1^

|  | **Dietary pattern 1** | | | | | **Dietary pattern 2** | | | | |
| --- | --- | --- | --- | --- | --- | --- | --- | --- | --- | --- |
| **Outcomes** | **Q1** | **Q2** | **Q3** | **Q4** | ***P**** | **Q1** | **Q2** | **Q3** | **Q4** | ***P**** |
|  |  |  |  |  |  |  |  |  |  |  |
| Total Fat *%TE*^§^ | 35.9 (0.6) | 35.8 (0.6) | 35.6 (0.6) | 34.0 (0.6) | 0.100 | 38.2 (0.6)^a^ | 35.8 (0.6)^ab^ | 34.2 (0.6)^bc^ | 33.1 (0.6)^c^ | <0.001 |
| SFA *%TE*^§^ | 13.2 (0.3)^a^ | 13.1 (0.3)^a^ | 12.7 (0.3)^ab^ | 11.5 (0.3)^b^ | <0.001 | 13.2 (0.3) | 12.8 (0.3) | 12.4 (0.3) | 12.0 (0.3) | 0.184 |
| MUFA *%TE*^§^ | 13.1 (0.3) | 12.9 (0.3) | 13.0 (0.3) | 12.7 (0.3) | 0.358 | 14.2 (0.3)^a^ | 12.9 (0.3)^ab^ | 12.5 (0.3)^b^ | 12.1 (0.3)^b^ | <0.001 |
| n-3 PUFA *%TE*^§^ | 0.77 (0.04) | 0.79 (0.04) | 0.86 (0.04) | 0.83 (0.04) | 0.239 | 0.90 (0.04)^a^ | 0.90 (0.04)^a^ | 0.73 (0.04)^a^ | 0.73 (0.04)^a^ | 0.002 |
| n-6 PUFA *%TE*^§^ | 4.2 (0.2)^a^ | 4.4 (0.2)^ab^ | 4.9 (0.2)^ab^ | 5.0 (0.2)^b^ | <0.001 | 5.3 (0.2)^a^ | 4.7 (0.2)^ab^ | 4.3 (0.1)^b^ | 4.2 (0.2)^b^ | <0.001 |
| CHO *%TE* | 42.8 (0.7)^a^ | 44.3 (0.7)^ab^ | 44.6 (0.7)^ab^ | 46.9 (0.7)^b^ | 0.001 | 39.8 (0.7)^a^ | 43.8 (0.7)^b^ | 46.3 (0.6)^bc^ | 48.8 (0.7)^c^ | <0.001 |
| Total Sugars *%TE* | 15.6 (0.5)^a^ | 17.8 (0.5)^b^ | 18.9 (0.5)^b^ | 21.3 (0.5)^c^ | <0.001 | 18.3 (0.5) | 18.4 (0.5) | 18.0 (0.5) | 18.9 (0.5) | 0.602 |
| Free sugars *%TE* | 11.7 (0.4)^a^ | 11.4 (0.4)^ab^ | 10.6 (0.4)^ab^ | 10.0 (0.4)^b^ | 0.007 | 9.9 (0.4)^a^ | 10.8 (0.4)^ab^ | 10.9 (0.4)^ab^ | 12.2 (0.4)^b^ | 0.001 |
| Fibre (AOAC) *g/d*^§^ | 17.4 (0.5)^a^ | 22.2 (0.5)^b^ | 24.4 (0.5)^c^ | 28.3 (0.5)^d^ | <0.001 | 24.4 (0.6) | 23.6 (0.5) | 22.9 (0.5) | 21.5 (0.6) | 0.016 |
| Protein *%TE*^§^ | 17.0 (0.3) | 17.0 (0.3) | 17.2 (0.3) | 17.3 (0.3) | 0.568 | 18.7 (0.3)^a^ | 17.1 (0.3)^b^ | 16.6 (0.3)^b^ | 16.1 (0.3)^b^ | <0.001 |
| Sodium *mg/d*^§^ | 2682 (62)^a^ | 2562 (60)^ab^ | 2470 (60)^ab^ | 2304 (62)^b^ | <0.001 | 2425 (62) | 2499 (61) | 2539 (60) | 2556 (64) | 0.173 |
| Alcohol *g/d*^§^ | 13.9 (1.0)^a^ | 8.9 (1.0)^ab^ | 6.8 (1.0)^b^ | 5.1 (1.0)^b^ | <0.001 | 10.3 (1.0) | 10.1 (1.0) | 8.7 (1.0) | 5.7 (1.0) | 0.274 |
| AHEI-2010 | 46.8 (0.9)^a^ | 58.1 (0.9)^b^ | 63.5 (0.9)^c^ | 69.4 (0.9)^d^ | <0.001 | 63.4 (1.1)^a^ | 62.8 (1.0)^a^ | 56.2 (1.0)^b^ | 56.0 (1.1)^b^ | <0.001 |

^1^ Participants’ nutrient intakes are expressed as estimated marginal means (standard error) stratified by the quartiles of adherence to the dietary pattern.

^§^ Log10 transformed prior to statistical analysis. Since alcohol included g/d values of 0, a constant of 1 was added prior to log10 transformation. Estimated marginal means are shown for the untransformed data.

***** p≤0.01 was considered significant for ANCOVA after adjusting for covariates (sex, age, menopausal status, supplement usage, energy intake (fibre and sodium only), and cardiovascular disease risk) and pairwise comparisons with Bonferroni correction. Different superscript letters ^(a,b,c,d)^ represent significant differences between quartile groups.

^†^ Pairwise comparisons were not significantly different between quartile groups.

Abbreviations: AHEI, Alternative Healthy Eating Index; AOAC, Association of Analytical Chemists; CHO, carbohydrates; MUFA, monounsaturated fatty acids; PUFA, polyunsaturated fatty acids; SFA, saturated fatty acids; TE, total energy.
